# Construction of a high-density genetic linkage map and QTL mapping for bioenergy-related traits in sweet sorghum [*Sorghum bicolor* (L.) Moench]

**DOI:** 10.3389/fpls.2023.1081931

**Published:** 2023-06-05

**Authors:** Birgul Guden, Engin Yol, Cengiz Erdurmus, Stuart James Lucas, Bulent Uzun

**Affiliations:** ^1^ Department of Field Crops, Faculty of Agriculture, Akdeniz University, Antalya, Türkiye; ^2^ Department of Field Crops, West Mediterranean Agricultural Research Institute, Antalya, Türkiye; ^3^ Sabanci University Nanotechnology Research and Application Centre, Sabanci University, Istanbul, Türkiye

**Keywords:** bioethanol, cereal, ddRAD-seq, pyramiding, QTL

## Abstract

Sorghum is an important but arguably undervalued cereal crop, grown in large areas in Asia and Africa due to its natural resilience to drought and heat. There is growing demand for sweet sorghum as a source of bioethanol as well as food and feed. The improvement of bioenergy-related traits directly affects bioethanol production from sweet sorghum; therefore, understanding the genetic basis of these traits would enable new cultivars to be developed for bioenergy production. In order to reveal the genetic architecture behind bioenergy-related traits, we generated an F_2_ population from a cross between sweet sorghum cv. ‘Erdurmus’ and grain sorghum cv. ‘Ogretmenoglu’. This was used to construct a genetic map from SNPs discovered by double-digest restriction-site associated DNA sequencing (ddRAD-seq). F_3_ lines derived from each F_2_ individual were phenotyped for bioenergy-related traits in two different locations and their genotypes were analyzed with the SNPs to identify QTL regions. On chromosomes 1, 7, and 9, three major plant height (PH) QTLs (*qPH*1.1, *qPH*7.1, and *qPH*9.1) were identified, with phenotypic variation explained (PVE) ranging from 10.8 to 34.8%. One major QTL (*qPJ*6.1) on chromosome 6 was associated with the plant juice trait (PJ) and explained 35.2% of its phenotypic variation. For fresh biomass weight (FBW), four major QTLs (*qFBW*1.1, *qFBW*6.1, *qFBW*7.1, and *qFBW*9.1) were determined on chromosomes 1, 6, 7, and 9, which explained 12.3, 14.5, 10.6, and 11.9% of the phenotypic variation, respectively. Moreover, two minor QTLs (*qBX*3.1 and *qBX*7.1) of Brix (BX) were mapped on chromosomes 3 and 7, explaining 8.6 and 9.7% of the phenotypic variation, respectively. The QTLs in two clusters (*qPH*7.1/*qBX*7.1 and *qPH*7.1/*qFBW*7.1) overlapped for PH, FBW and BX. The QTL, *qFBW*6.1, has not been previously reported. In addition, eight SNPs were converted into cleaved amplified polymorphic sequences (CAPS) markers, which can be easily detected by agarose gel electrophoresis. These QTLs and molecular markers can be used for pyramiding and marker-assisted selection studies in sorghum, to develop advanced lines that include desirable bioenergy-related traits.

## 1 Introduction

Bioenergy is defined as energy obtained from biomass or its metabolic by-products (Calvin et al., 2021). Taking into consideration current global warming, climate change and energy security issues, it has important advantages over conventional fossil fuels (Prasad et al., 2021; Rather et al., 2022). Sweet sorghum [*Sorghum bicolor* (L.) Moench], as a first-generation energy crop (Yadav et al., 2019), offers a valuable potential source of sustainable bioenergy production (Olivera et al., 2020) due to its high water-use efficiency and resistance to heat and drought conditions (Abebe et al., 2020). Sorghum is also considered a “failsafe” in agroecosystems (Paterson, 2008), due to its resilience and productivity in conditions that are too hot and dry for most other cereals. Sweet sorghum has drawn attention not only for the production of bioenergy from directly fermentable sugars contained in its stalk, but also for its high biomass production capacity (Smith et al., 1987; Murray et al., 2009). Compared to other bioenergy sources such as sugarcane, sweet sorghum has a comparatively low input requirement, high water use efficiency, drought resistance and high yield, and furthermore also produces grain that can be used in food or feed; therefore, it is considered a better alternative for bioethanol production from stem sugars, as both energy and food can be produced simultaneously (Reddy et al., 2005).

Selection for bioenergy producing sorghum cultivars depends on the characterization of biomass-related traits like plant height, sugar content, and fresh biomass. Plant height is an important part of plant architecture (Guden et al., 2020a) that has a significant correlation with biomass production (Calvino and Messing, 2012). Because quantitative trait loci (QTL) for biomass have been identified to be co-localized with height QTLs (Brown et al., 2008), breeders usually target taller genotypes in their sorghum biomass development strategy. The Brix value, which also has a positive correlation with height (Wang et al., 2020a), is an indicator of the total sugar content in stem juice and so represents an important bioenergy trait. Extractable juice content, which can be directly fermented into ethanol, is another trait that contributes greatly to high bioenergy production (Takaki et al., 2015). Considering all these factors, recent breeding studies have focused on crop improvement by identifying and selecting for QTLs that control these traits (Regassa and Wortmann, 2014; Mace et al., 2019). Employing quantitative genetics and molecular-based technologies, several previous mapping studies have been conducted to determine associations between gene/QTL regions and bioenergy-related traits (Kajiya-Kanegae et al., 2020; de Souza et al., 2021). de Souza et al. (2021) used 272 RIL plants from a cross between two sweet sorghum lines (Brandes × Wray) to create a genetic map and mapped 33 QTLs for bioenergy traits, 14 of which were found in all three environments tested. Jin et al. (2021) constructed a genetic map and identified a total of 43 QTLs for biomass traits, several of which were stable across 2 years. However, the high variability of agronomic traits between different locations and years demands further studies, in order to identify stable QTLs that consistently improve traits in multiple environments and genetic backgrounds (Chen et al., 2020).

Single Nucleotide Polymorphism (SNP) markers have higher coverage in plant genomes than other marker types such as RFLP and SSRs (Kumar et al., 2009) and can be identified rapidly using next generation sequencing methods such as whole-genome sequencing, genotyping-by-sequencing (GBS), restriction site-associated DNA sequencing (RAD-seq), double digest restriction site-associated DNA sequencing (ddRAD-Seq), and specific-locus amplified fragment sequencing (SLAF-seq). Among these, ddRAD-seq is a popular and cost-effective method for SNP discovery that includes digesting the DNA with two defined restriction enzymes, pseudo-random sampling, and size-selection of fragments for sequencing (Peterson et al., 2012). It is inexpensive compared to whole-genome sequencing, and as many restriction sites are conserved between different genotypes, no reference genome is required for bioinformatic analysis (Andrews et al., 2016). The application of ddRAD-seq has been reported in many species, such as apple (*Malus domestica* Borkh.) (Ma et al., 2017), orchid (*Orchis* spp.) (Roy et al., 2017), onion (*Allium cepa* L.) (Lee et al., 2018), sesame (*Sesamum indicum* L.) (Basak et al., 2019) and hazelnut (*Corylus avellana* L.) (Oztolan-Erol et al., 2021). However, ddRAD-seq has not been applied in sorghum to date.

The objectives of this study were to (i) construct a genetic map for sorghum using ddRAD-seq, (ii) identify QTLs for bioenergy-related traits, and (iii) develop user-friendly PCR-based SNP markers and validate them on diverse genetic sources.

## 2 Materials and methods

### 2.1 Plant materials and experimental design

An F_2_ population, consisting of 175 individuals, was obtained from a cross between the cultivars Erdurmus and Ogretmenoglu, registered by the Western Mediterranean Agricultural Research Institute of Turkey (WMARI). Erdurmus (♀) is a sweet sorghum cultivar with high plant height, juice and Brix content. Moreover, it is known to have a high bioethanol yield (Guden et al., 2020b) and was used as the female parent. The male parent differs from the female cultivar in its bioenergy-related traits. Ogretmenoglu (♂) is a grain sorghum cultivar that has short plant height, low plant juice, low Brix content, and low bioethanol yield (Guden et al., 2020b). The parents were crossed in a greenhouse in 2016 and F_1_ plants were selfed in the experimental fields of Akdeniz University (36°53′ N, 38°30′ E and altitude of 15 m) in 2017. In the subsequent year, the F_2_ population was grown at the WMARI (36° 52′ N and 30° 50′ E, 41 m above sea level), Antalya, Turkey. Individual lines derived from the F_2_ population were advanced to F_3_ and this population was grown in two different locations, which are the WMARI in Antalya (lowland) and the Soil, Water and Deserting Control Research Institute in Konya (highland) (37° 48’ N and 32° 30’ E, 1072 m above sea level), using a randomized complete block design with three replicates, in the 2019 growing season. The climatic data for the fields is presented in **Table 1**. Plants were sown in two rows with a length of 5 m at a spacing of 0.7 m between rows and a plant spacing of 0.2 m. At both locations, chemical fertilizers were applied prior to seedling germination at rates of 60 kg/ha N and 60 kg/ha P_2_O_5_. A further application of 60 kg/ha N was added once the plants’ height reached 50 cm.

**Table 1:** Monthly temperature and rainfall values in the sorghum growing period of 2019.

Months	Lowland						Highland					
	Temperature (°C)				Rainfall (mm)		Temperature (°C)				Rainfall (mm)	
	Minimum	Maximum	Average (2019)	Long Term Average*	Average (2019)	Long Term Average*	Minimum	Maximum	Average (2019)	Long Term Average*	Average (2019)	Long Term Average*
**May**	9.9	36.3	21.3	21.8	7.0	29.9	2.9	34.1	18.5	15.7	13.2	43.3
**June**	12.2	39.7	25.8	25.6	13.0	9.7	8.2	33.0	21.2	20.2	30.4	24.3
**July**	16.0	40.9	28.6	28.8	0.0	2.9	10.1	34.9	22.4	23.6	6.2	6.6
**August**	16.1	42.8	28.7	27.8	0.0	2.9	10.9	34.8	22.7	23.1	7.8	5.3
**September**	12.1	36.4	25.2	24.3	77.0	12.9	2.3	31.6	18.5	18.6	9.6	11.8

*Average of 1953- 2019.

### 2.2 Phenotypic observations

Bioenergy-related traits including plant height, plant stalk juice weight, Brix and fresh biomass weight were used to characterize plants from the F_3_ populations in both locations. Five plants representing each plot were selected and harvested manually. Plant height (PH) was identified as the distance between the ground and the top of the flag leaf after full panicle emergence (cm). The fresh biomass weight (FBW) was identified by cutting the stem of each plant just above ground level and weighing immediately (g). The other two observations were performed after the panicles, leaves, and leaf sheaths of the plants were removed. Plant juice (PJ), obtained by pressing the stems with a roller mill, was accumulated in a graduated cylinder. Juice content was calculated as % of total biomass using the formula: [juice weight (g)/biomass weight (g)] x 100. Brix (BX) was measured with a digital refractometer (Hanna, HI96801, USA) using the extracted juices (%).

### 2.3 Statistical analysis

The correlation analyses and analysis of variance (ANOVA, PROC GLM) were performed with Past 4.03 and SAS 9.0 software, respectively. Broad-sense heritability ${(}_{\text{h}}^{\text{b}-\text{s}}2)$ (Allard, 1960) of all traits was calculated by:


$${\text{h}}_{\text{b}-\text{s}}^{2}=\left[\left(\sigma^{2}G\right)/\left(\sigma^{2}P\right)\right]\text{ x }100$$

where: ${\text{h}}_{\text{b}-\text{s}}^{2}$ ; heritability in broad sense, σ^2^ G; genotypic variance and, σ^2^ P; phenotypic variance.

### 2.4 DNA extraction and sequencing

Young leaves from 175 F_2_ plants and their parents were collected and stored at -80°C. Genomic DNA was extracted with a modified CTAB method (Doyle and Doyle 1990). The modification was accomplished by adding ethanol and isopropanol, which were utilized to precipitate DNA. DNA concentration was measured with a Qubit 2.0 Fluorometer using a Qubit DNA Assay Kit (Life Technologies, Thermo Fisher Scientific), while agarose gel electrophoresis was used to check for degradation.

A modified version of the ddRAD-seq method was adapted for sequencing library construction. Briefly, all the DNAs from F_2_ lines and their parents were digested with the endonucleases *Vsp*I (ATTAAT) and *Msp*I (CCGG) for 2 hours at 37°C. The double-digested samples were cleaned with Ampure XP beads (Beckman Coulter Genomics) followed by ligation with barcoded P1 and P2 adaptors using T4 DNA ligase buffer (Peterson et al., 2012). The P1 adapter’s 3’ end was modified to match the overhanging *Vsp*I restriction cut site, because of the selection of six-base cutter *Vsp*I instead of *Eco*RI, which is the enzyme used in the original ddRAD protocol. Following ligation, each reaction was subjected to PCR amplification (15 cycles) using genotype-specific indexed PCR primers. Amplified fragments were checked on an agarose gel and a size window of 400-500 bp fragments was selected. The selected products from each sample were pooled, and the resulting ddRAD-seq genomic library was sequenced using the Illumina Hi-Seq platform *via* 150-bp paired-end sequencing.

### 2.5 SNP calling

Single-nucleotide polymorphism (SNP) calling was performed using the following steps: the raw reads were demultiplexed with Je (v1.2.1) (Girardot et al., 2016). Adapters and restriction enzyme sequences were trimmed with the use of fastp (v0.20.1) (Chen et al., 2018), while bases were marked as ‘qualified’ if they had a phred-scale quality score >15; this parameter was used to discard low quality reads with >40% ‘unqualified’ bases. The other filtering options used were: minimum read length of 15, low complexity sequences comprise no more than 30% of read length; reads failing these thresholds were eliminated. Clean sequences of each individual were aligned to the sorghum BTx623 version 3 reference genome (McCormick et al., 2018) using Bowtie2 (v2.3.4.3) (Langmead and Salzberg, 2012) with default parameters. The individual BAM files were analyzed using freebayes (v13.1) (Garrison and Marth, 2012) with a simple diploid calling model and filtering. A minimum coverage value of 20 was used for the identification of sequence variants. To combine each individual variant file, Bcftools merge (v1.10) (Li et al., 2009) was used. This file was converted to BED (browser extensible data) (Quinlan and Hall, 2010) format for the purpose of storing the coordinates of the genomic regions that contained putative variants. After that, in order to improve detection of uncommon alleles, the previously created BAM files were re-analyzed with freebayes using 10x minimum coverage but limiting matches to regions included in the BED files, in order to identify reliable SNP alleles for each genotype. Then all variant files were merged into a single variant file. Putative SNPs were filtered with the following parameters: minimum site count 80, and minimum–maximum heterozygous proportion 0.3-0.8.

### 2.6 Construction of linkage map

Polymorphic SNPs identified between the parental lines were converted to ABH genotype format using Tassel V5.2.52 (Glaubitz et al., 2014). Sites with high missing data (> 20%) and segregation distortion (Chi-square, p< 0.001) were filtered out, and a genetic linkage map was constructed with the JoinMap 4.1 program (Van Ooijen, 2011). The linkage map was constructed with a minimum logarithm of odds (LOD) score of 10 and a maximum recombination fraction of 0.2 was estimated for the marker groups. The recombination rate was converted into map distance in cM (centiMorgans) by the Kosambi mapping function (Kosambi, 1944). We compared the genetic map to the reference genome (as a representative physical map) to determine alignment between the genome and the genetic map, and dot plots were created with Excel. We used a Multiple QTL Mapping (MQM) function which was equivalent to Composite Interval Mapping (CIM) approach to detect QTLs for bioenergy-related traits in MapQTL 6 (Van Ooijen, 2009) with combined phenotypic data. MapChart 2.32 (Voorrips, 2002) was used for the mapping of the positions of QTLs with LOD scores of at least 4.0 for all traits except BX (LOD ≥ 3.0).

### 2.7 Development of CAPS markers

We designed cleaved amplified polymorphic sequence (CAPS) markers to be associated with the bio-energy related trait loci identified in the F_2_ population. Flanking sequences with the criteria of predicted product size ranging from 200 to 500 bp either side of putative SNPs were transferred to the Primer3 program (Untergasser et al., 2012) and suitable markers containing the putative SNPs were developed. The suitable restriction endonucleases for these SNP regions were determined using NebCutter (Vincze et al., 2003).

These markers were validated in diverse genetic backgrounds including eight sweet sorghum lines (47, 69, 91, 325, 424, 456, 532, and 507), parental lines (Erdurmus and Ogretmenoglu) and one sweet sorghum cultivar (Uzun). PCR was performed in 15-μL PCR mixtures containing 100 ng genomic DNA, 1.5 μl 10 × PCR buffer, 2.5 mM MgCl2, 1.25 μL dNTP mix, 1 μL each of forward and reverse primers (10 pmol), 0.2 μL Taq DNA polymerase (5 U/ml), 1.5 μL genomic DNA, and Milli-Q water. Thermocycling started with 94°C initial denaturation for 5 min, and was followed by 30 cycles of 94°C denaturation for 30 s, 55°C annealing for 30 s, 72°C extension for 40 s, and 72°C final extension for 10 min. The amplicons were digested with the appropriate restriction enzyme identified from the SNP region for each primer (New England BioLabs, Beijing, China) in 21 μL reaction containing 10 μL PCR product, 2 μL 10 × NEBuffer, 1 μL restriction enzyme, and Milli-Q water, with the enzyme specific temperature incubated for 3 h. Finally, after the products were separated by electrophoresis on a 2.5% agarose gel, they were visualized with UV light.

## 3 Results

### 3.1 Phenotypic variation in bioenergy-related traits

Significant differences were recorded for all the bioenergy-related traits between genotypes and in both environments (**Table 2**). The plants grown in the highland location exhibited less variation than lowland for PH and FBW. The ANOVA showed that the variation within the F_3_ population between genotypes (σg_2_) and environments (σE^2^) were significantly different for all traits and locations, but the variation of the genotype × environment interaction (σGxE^2^) was only statistically significant (P< 0.01) for PH. Coefficients of variation (CV) for each trait ranged from 11.05 for PH at the highland site up to 30.35 for FBW at the lowland site. Across the environments, PH had the lowest CV, and FBW had the highest. Moreover, the broad-sense heritability for PH, PJ, and FBW were 0.87, 0.67, and 0.80, respectively, showing that genotypic variance accounted for a high proportion of the phenotypic variation. Correlation analysis indicated that PH was significantly and positively correlated with FBW and PJ with correlation coefficients of 0.54 and 0.30, respectively. Similarly, a significant positive correlation was observed between FBW and PJ (0.30), while BX was not significantly correlated with the other traits (**Table 3**).

**Table 2:** Phenotypic variation for bioenergy-related traits in F_3_ population.

Traits	Environments										Across the Environments					
	Lowland					Highland										
	Min	Max	Mean	σg^2a^	CV	Min	Max	Mean	σg^2a^	CV	Mean	σgxE^2b^	σg^2a^	CV	σE^2c^	${\text{h}}_{\text{b}-\text{s}}^{2}$ (%)
**PH (cm)**	101.33	298.67	202.80	4.09**	13.27	102.33	325.00	225.00	9.03**	11.05	214.00	1.44**	11.30**	12.11	208.59**	0.87
**BX (%)**	4.15	17.55	10.66	1.70**	14.41	4.25	13.75	8.44	1.86**	16.52	9.55	1.26	2.29**	15.36	620.09**	0.44
**PJ (%)**	11.00	82.00	34.24	1.49**	17.17	8.00	74.00	35.02	3.46**	14.80	34.63	1.17	3.54**	16.01	5.25*	0.67
**FBW (g)**	30.00	1068.00	382.80	2.47**	30.35	46.00	940.00	440.34	5.05**	21.73	411.57	1.13	5.89**	25.87	76.05**	0.80

*significant at p ≤0.05.

**significant at p ≤ 0.01.

a Genotype variance.

b Genotype by environment variances for two locations.

c Environment variance ${\text{h}}_{\text{b}-\text{s}}^{2}$ Broad-sense heritability.

**Table 3:** Pearson correlation coefficients between traits across two environments.

Traits	PH	BX	PJ
**BX**	0.28		
**PJ**	0.30**	0.08**	
**FBW**	0.54**	-0.02	0.30**

PH, Plant height; BX, Brix; PJ, Plant juice; FBW, Fresh biomass weight.

**significant at p ≤ 0.01.

### 3.2 Genotyping

A total of 177 individuals, including 175 F_2_ plants and two parents, were used in construction of the ddRAD-seq library. After quality filtering, 429.23 M reads were obtained for the analyses. The collection was represented by a mean of 2.425 M reads per genotype and average 41.17% guanine-cytosine (GC) content. The highest read counts and guanine-cytosine (GC) content were 5.96 M and 51.16% for the genotypes 147 and 171, respectively.

### 3.3 Linkage map construction

**Figure 1:**
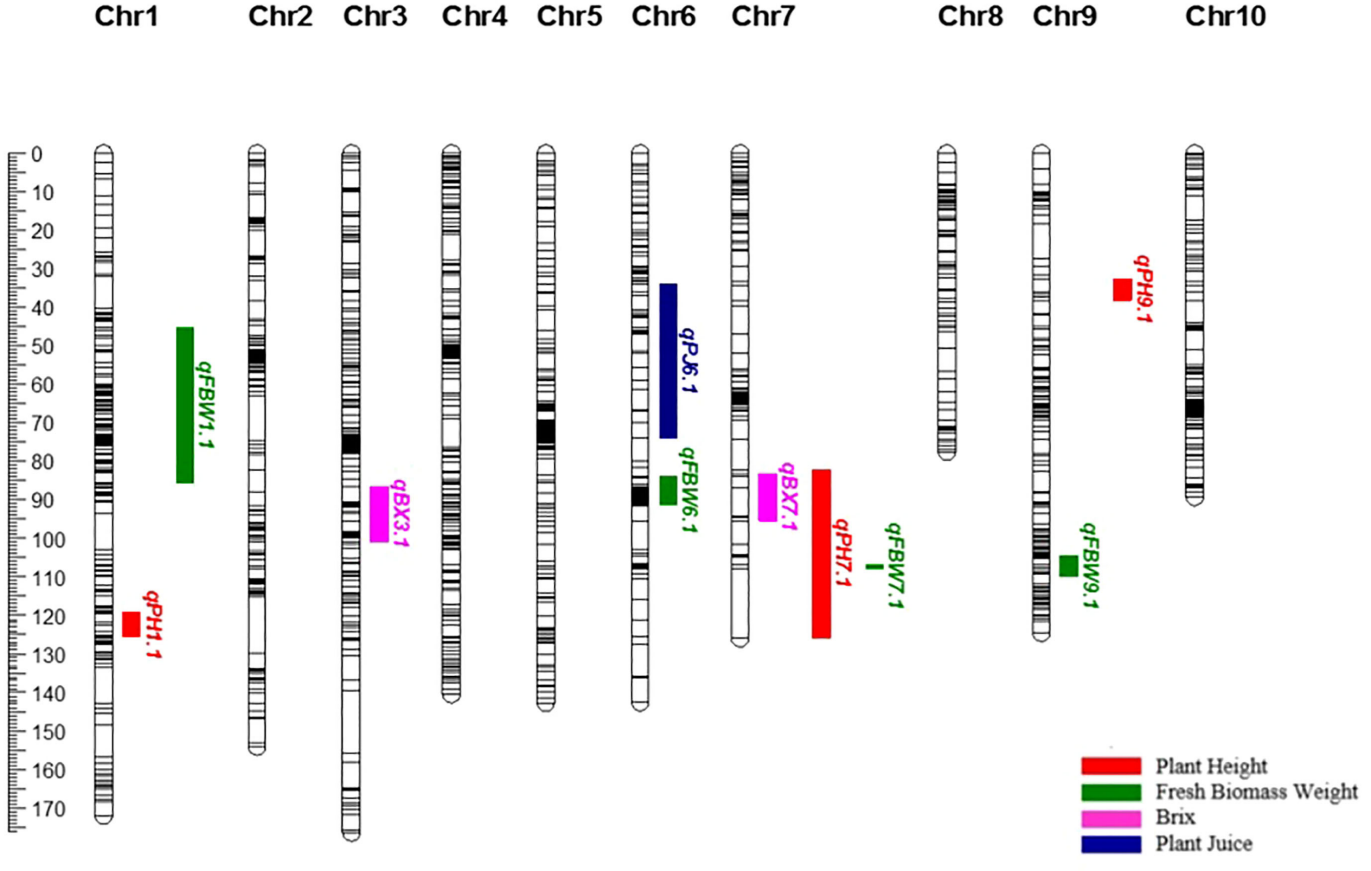
Genetic map, showing the locations of QTLs for bioenergy-related traits on chromosomes 1, 3, 6, 7 and 9.

The SNPs called from the 175 F_2_ individuals were used to create the genetic map. After filtering, 1438 SNPs were assigned to 10 linkage groups (LGs) (**Figure 1**). The total length of the map was 1349.72 cM with an average marker interval of 0.94 cM. The length of each LG ranged from 77.46 cM to 176.36 cM. Among the LGs, the greatest number of markers was determined on LG3, which contained 193 markers with an average distance of 0.91 cM between markers. Conversely, LG8 had the fewest markers (86) with an average distance of 0.90 cM (**Table 4**). The largest gap was 17.98 cM in LG7, followed by gaps of 14.66 and 9.49 cM in LG2 and LG1, respectively. The LGs were then compared with the SNP positions mapped on to the *S. bicolor* BTx623 reference genome as a surrogate physical map (**Figure S1**). For most linkage groups, a highly consistent marker order was observed towards the end of the chromosome arms, but with some scattering in the middle of each chromosome. This is an expected result due to the low recombination rate in peri-centromeric regions, meaning that there have been too few recombination events to accurately resolve the marker order in the F_2_ population. Interestingly, a series of SNPs on LG8 and a second on LG10 mapped on the other side of the centromere in the Erdurmus x Ogretmenoglu population compared to the reference genome. This may indicate the presence of chromosome translocations in one of these cultivars, although further physical mapping of the parental lines would be required to confirm this.

**Table 4:** SNP distribution on each chromosome.

Linkage Groups	Total Genetic Distance (cM)	Number of Markers (SNPs)	Average Distance/SNP (cM)
**LG 1**	171.98	185	0.92
**LG 2**	154.11	150	1.02
**LG 3**	176.36	193	0.91
**LG 4**	145.04	178	0.81
**LG 5**	142.71	161	0.88
**LG 6**	142.46	113	1.26
**LG 7**	125.86	16	1.08
**LG 8**	77.46	86	0.90
**LG 9**	124.46	138	0.90
**LG 10**	89.24	118	0.69
**Total**	1349.72	1438	0.94

### 3.4 QTL analysis for bioenergy-related traits

A total of ten QTLs were identified for all the traits (**Table 5**) (**Figure 1**). These are as follows:

**Table 5:** QTL mapping for bioenergy-related traits in F_3_s.

Trait	QTL Name	Linkage Group	Position (cM)	Peak Marker	LOD	PVE (%)	*Dominant Effect	*Additive Effect	Source of Favorable Parental Allele
**PH**	***qPH*1.1**	1	119.296-119.661	Chr1_64500453- Chr1_64555162	4.34	10.8	14.36	13.64	E
	***qPH*7.1**	7	94.693	Chr7_59610235	16.23	34.8	10.66	29.33	E
	***qPH*9.1**	9	38.245	Chr9_3610075	5.50	13.5	-2.23	20.07	O
**BX**	***qBX*3.1**	3	93.037	Chr3_55215755	3.42	8.6	-0.006	0.37	E
	***qBX*7.1**	7	84.046	Chr7_57982334	3.90	9.7	-0.003	0.41	E
**PJ**	***qPJ*6.1**	6	55.681	Chr6_ 50628405	16.46	35.2	0.08	3.51	O
**FBW**	***qFBW*1.1**	1	69.221	Chr1_25475915	5.0	12.3	55.26	-38.53	O
	***qFBW*6.1**	6	86.462	Chr6_39024006	5.95	14.5	40.91	52.25	E
	***qFBW*7.1**	7	106.748	Chr7_62075852	4.27	10.6	2.22	48.11	E
	***qFBW*9.1**	9	108.114	Chr9_57098254	5.67	13.9	46.76	50.62	E

* Additive and Dominant effects: positive and negative values indicate that increased and decreased effect, respectively.

PH, Plant height; BX, Brix; PJ, Plant juice; FBW, Fresh biomass weight; E, Erdurmus; O, Ogretmenoglu.

PH: Three major QTLs were detected for PH on chromosomes 1, 7, and 9 (*qPH*1.1, *qPH*7.1, and *qPH*9.1). The logarithm of the odds (LOD) scores ranged from 4.34 to 16.23, and the percentage of phenotypic variation explained by each QTL ranged from 10.80 to 34.80%. The largest PVE, 34.8%, was for *qPH*7.1 at the position of 94.693 cM on LG7. The positive effects of *qPH*1.1 and *qPH*7.1 were conferred by the allele from Erdurmus, while the advantageous allele for the other QTL, *qPH*9.1, was from Ogretmenoglu.

**Figure 2:**
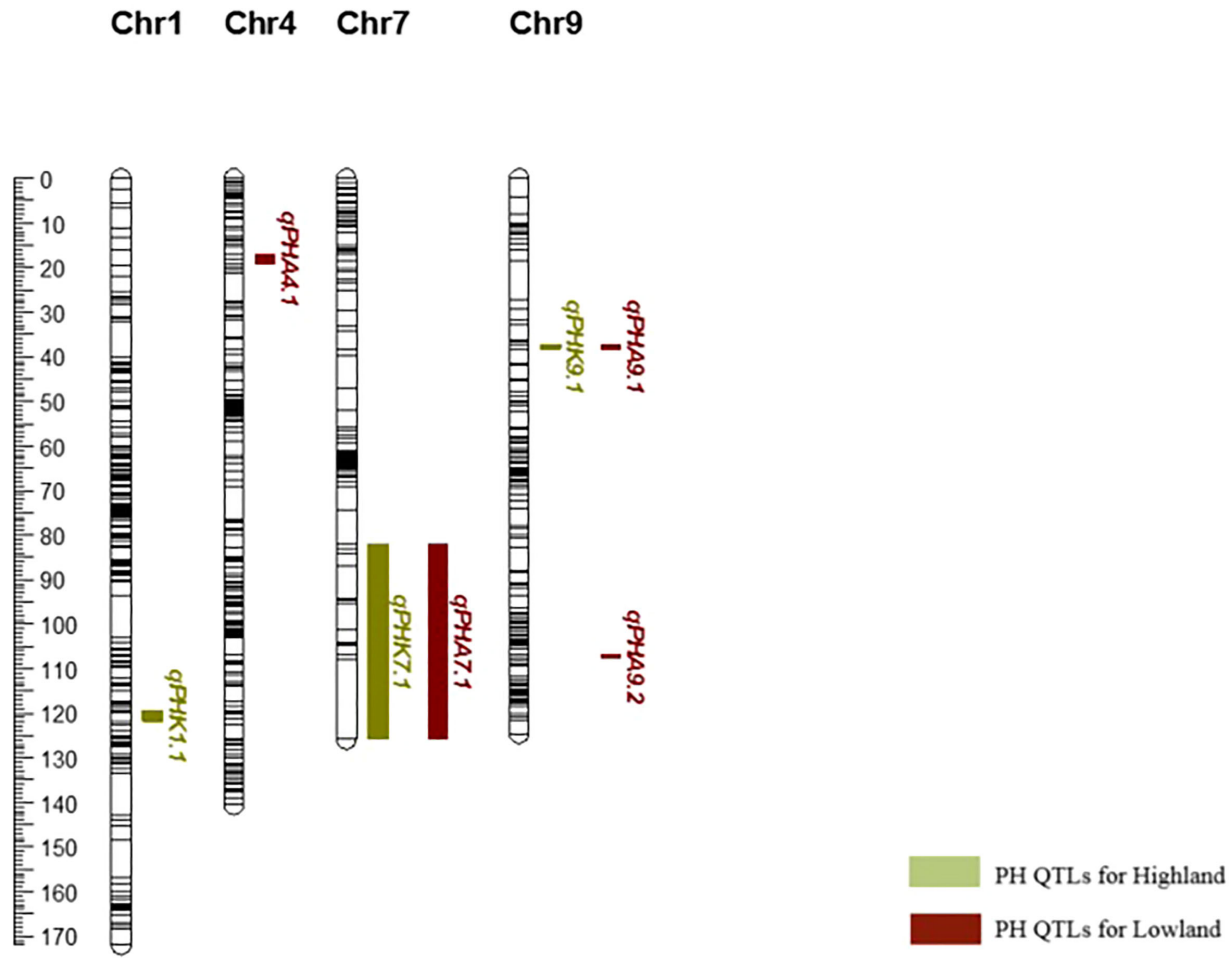
Genetic map of chromosomes 1, 4, 7 and 9 showing plant height QTLs determined individually for each environment.

After it was determined that GxE was significant for PH, QTLs were re-analyzed for the two environments, individually. For highland, three major QTLs were determined for PH on chromosomes 1, 7, and 9 (*qPHK*1.1, *qPHK*7.1, and *qPHK*9.1), which are in the same location with the QTL obtained with combined data. For lowland, four major QTLs (*qPHA*4.1, *qPHA*7.1, *qPHA*9.1 and *qPHA*9.2) were identified for PH. One QTL, *qPHA*4.1, was located on chromosome 4, and the LOD score and PVE were determined to be 5.11 and 12.6%, respectively. Two major QTLs, *qPHA*7.1 and *qPHA*9.1, were determined on chromosomes 7 and 9, which are located in the same position with the QTLs identified with the combined data. Another major QTL, *qPHA*9.2, on chromosome 9, was identified at position 107.38 cM with a LOD score of 4.26 (**Figure 2**).

BX: Two minor QTLs were identified for BX, namely *qBX*3.1 and *qBX*7.1. These were located on chromosomes 3 and 7. Their LOD scores ranged from 3.42 to 3.90 and the PVE values ranged from 8.6 to 9.7%. *qBX*7.1 showed the highest PVE value with 9.7%. The positive effects of *qBX*3.1 and *qBX*7.1 were both contributed by Erdurmus.

PJ: One major QTL was detected for PJ (*qPJ*6.1) on chromosome 6. The PVE value and LOD score were determined to be 35.2% and 16.46, respectively. The positive effect on increased PJ was attributed to the Ogretmenoglu allele at *qPJ*6.1.

FBW: Four major QTLs were detected for FBW (*qFBW*1.1, *qFBW*6.1, *qFBW*7.1 and *qFBW*9.1) and these were located on chromosomes 1, 6, 7 and 9. The LOD scores and PVE values ranged from 4.27 to 5.95 and 10.6 to 14.5%, respectively. *qFBW*6.1 had the largest PVE of 14.5% and a positive effect from Erdurmus. The effect of increased FBW was attributed to Erdurmus at *qFBW*7.1 and *qFBW*9.1, whereas reduced FBW was attributed to the Ogretmenoglu allele at *qFBW*1.1.

### 3.5 Overlapping QTLs

**Figure 3:**
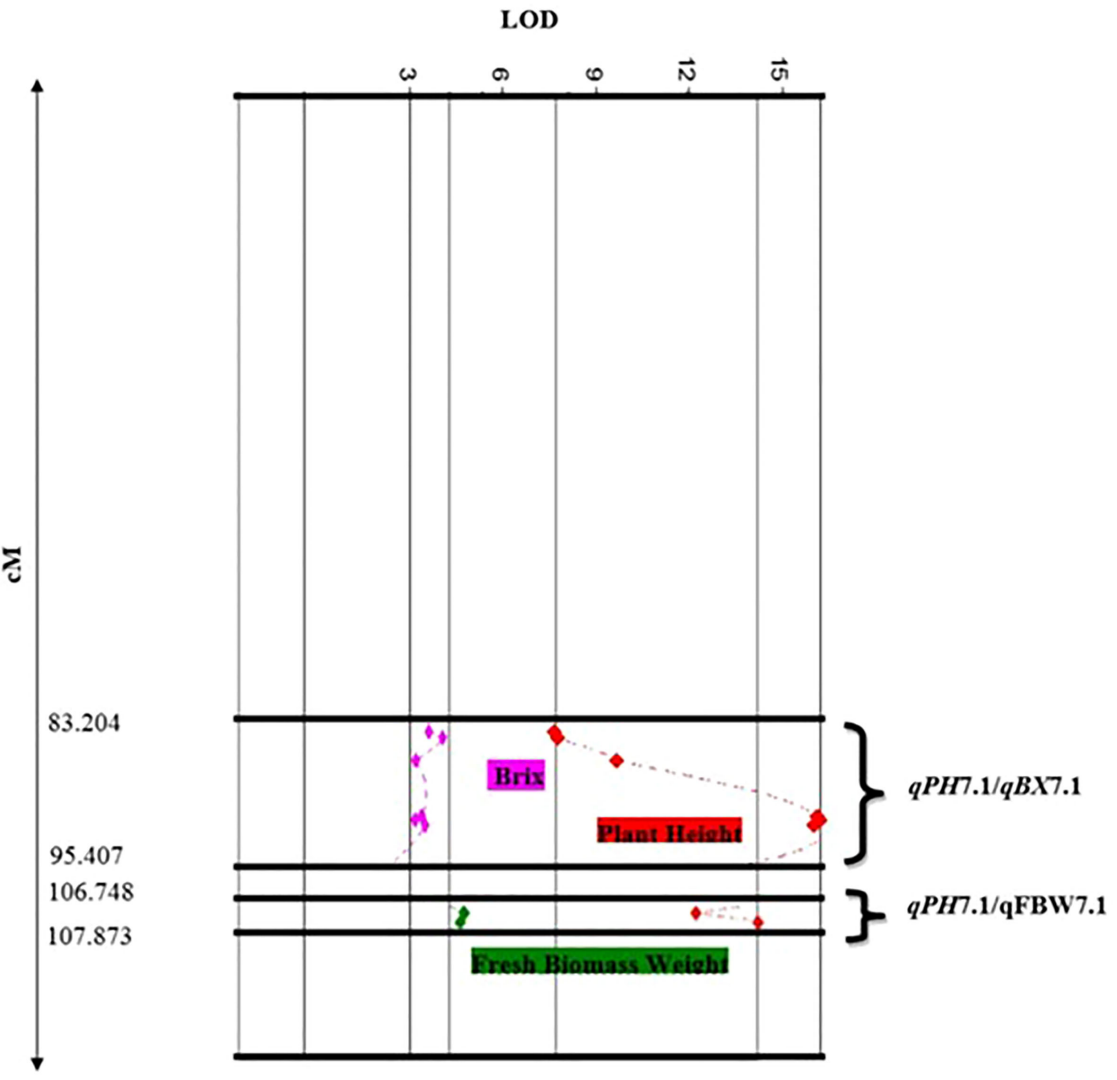
Close-up of QTL clusters showing LOD score distributions, for multiple traits with overlapping locations on the genetic map of chromosome 7.

In this study, we identified two clusters of overlapping QTLs. *qPH*7.1/*qFBW*7.1, and *qPH*7.1/*qBX*7.1 contained QTLs for PH, FBW, and BX on chromosome 7, which were located at 106.748-107.873 and 83.204-95.407 cM, respectively (**Figure 3**).

### 3.6 Marker development and validation

**Figure 4:**
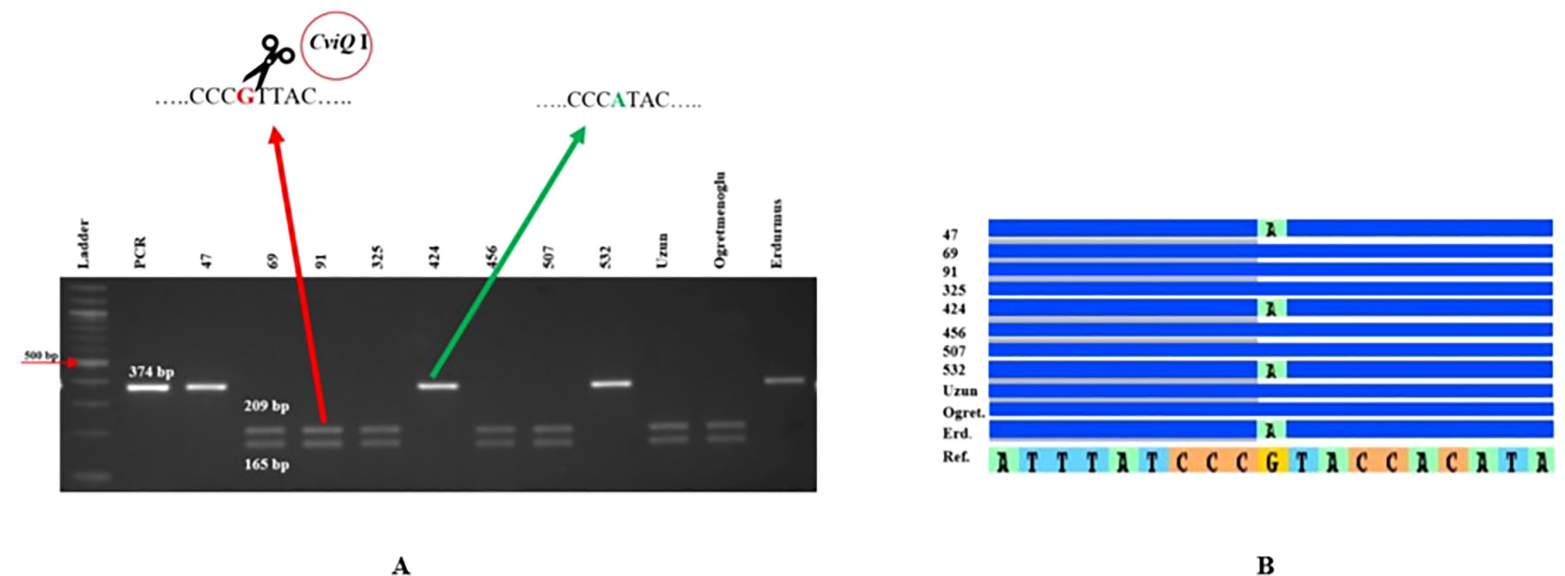
FBW5 CAPS marker validation. **(A)** Agarose gel electrophoresis for FBW5 after PCR amplification and *CviQ*I digestion. **(B)** Sequence alignment showing SNPs in the relevant region for the lines tested. Erd: Erdurmus, Ogret: Ogretmenoglu, Ref: Sorghum reference genome, 47, 69, 91, 325, 424, 456, 507, 532 and Uzun are lines and cultivar, respectively. Ladder: 100 bp markers, PCR: non-digested amplification of FBW5 locus.

Eight genome-specific CAPS markers associated with bioenergy-related traits were designed and developed. Forward and reverse sequences of the PCR primers for each marker, and their corresponding restriction enzymes, are given in **Table 6**. Eight sweet sorghum lines, one sweet sorghum cultivar and the two parents of our population were included in the validation. All amplified PCR and enzyme digested products generated fragments of the expected size for each CAPS marker. As an example, **Figure 4** shows validation of FBW5, a potential fresh biomass weight CAPS marker. Only seven individuals provided the band of the digested PCR product, which contained two fragments of 209 and 165 bp each, whereas other individuals presented the pattern of the non-digested PCR amplification, which was 374 bp. All eight markers were consistent with the expected results, showing perfect matches between SNPs and CAPS cleavage (**Table 7**). Moreover, the phenotype scores for bioenergy-related traits in these lines are also submitted in **Table 7**.

**Table 6:** Primer and enzyme combinations for genome-specific CAPS markers for the selected QTL-related regions.

Traits	Marker Name	Forward Primer	Reverse Primer	Restriction Enzyme
**PH**	**PH1**	TTTTGGGAGATCCTCCACTG	CGTGTGATTCAGCGTGGTAT	*SsP*I
	**PH7**	TGCATAGATCTAGTCATAAGAAACTCA	ATTAATGCACGGCCCATCT	*Tag*I
**BX**	**BX3**	TGGAGCAAAGACACATGGTAA	AAAGAATGCTGGAGTAAATGTGG	*Ssp*I
	**BX6**	TGACTCTGCCAAATCAGCAA	GCCCGTTGAATGGTCTAAACT	*Af*III
	**BX7**	GCAGGACAATGAAGCCCTTA	GGCTCGGATGCTCTAACAAG	*TaqI*
**PJ**	**PJ13**	TGATTCGTTTCCGAGTGTTG	TCACAAGTCAGGTGCTAAACC	*SexA*I
	**PJ15**	TCATACGTGGTAAGATCATATGAGAA	GAGGAATTAATTGGTGATGAAACA	*Hpy188*I
**FBW**	**FBW5**	GCCATATTAGAAAAGCATAGACAAA	GGGAAGTGATCCTTTTTAGTTGC	*CviQ*I

PH, Plant height; BX, Brix; PJ, Plant juice; FBW, Fresh biomass weight.

**Table 7:** CAPS marker validation and phenotypes in eight sweet sorghum lines, one sweet sorghum cultivar, and two parents.

Traits	Marker Name	Chr	Position	Ref	Lines/Cultivars										
					47	69	91	325	424	456	507	532	Uzun	Ogretmenoglu	Erdurmus
**PH**	**PH1**	7	59610262	C	**T**/T	**T**/T	**T/**T	C/C	C/C	C/C	C/C	**T**/T	C/C	C/C	**T**/T
	**PH7**	7	61486085	C	G/G	**C**/C	G/G	G/G	N/A*	**C**/C	**C**/C	G/G	**C**/C	**C**/C	G/G
**Phenotypes (cm)					300.0	279.0	290.5	261.5	326.5	292.0	280.5	326.4	302.0	139.8	296.0
**BX**	**BX3**	7	59610262	C	**T**/T	**T**/T	**T**/T	C/C	C/C	C/C	C/C	**T**/T	C/C	C/C	**T**/T
	**BX6**	3	55344886	T	**A/**A	T/T	T/T	T/T	T/T	T/T	T/T	T/T	T/T	**A**/A	T/T
	**BX7**	3	59803007	C	**T**/T	**T**/T	C/C	C/C	**T**/T	C/C	C/C	C/C	**T**/T	C/C	**T**/T
***Phenotypes (%)					15.04	14.44	12.15	14.61	14.40	11.43	13.81	11.58	12.49	12.86	15.24
**PJ**	**PJ13**	6	45662181	C	G/G	G/G	**C**/C	G/G	**C**/C	**C**/C	G/G	**C**/C	**C**/C	**C**/C	G/G
	**PJ15**	6	45972073	T	**G**/G	**G**/G	**G**/G	**G**/G	**G**/G	**G**/G	**G**/G	**G**/G	**G**/G	T/T	**G**/G
***Phenotypes (%)					32.34	19.77	23.93	25.60	14.16	30.30	12.22	10.13	22.61	14.49	31.86
**FBW**	**FBW5**	6	30373351	G	A/A	**G**/G	**G**/G	**G**/G	A/A	**G**/G	**G**/G	A/A	**G**/G	**G**/G	A/A
**Phenotypes (kg ha^-1^)**					4907	5603	8097	4187	4475	6955	3764	3672	7286	2091	6691

*N/A, No amplification.

G, Guanine; C, Cytosine; A, Adenine; T, Thymine; Ref, Sorghum reference genome.

** Guden et al., 2020a

*** Guden et al., 2020b; Uzun BSS496, Erdurmus BSS46.

Alleles marked in bold were cut by the related restriction enzyme.

## 4 Discussion

The ddRAD-Seq approach, a flexible diagnostic method for SNP-based genotyping, enabled the construction of a genetic map for an F_2_ population created by crossing high-sugar yielding (Erdurmus) and low-sugar yielding (Ogretmenoglu) sorghum genotypes. The obtained SNPs were distributed throughout the sorghum genome indicating the effectiveness of this technique, which made it possible to identify a large number of polymorphisms at a low cost (Peterson et al., 2012). The ddRAD-Seq technique has recently been utilized successfully in a variety of plants, including foxtail millet (Fukunaga et al., 2022), *Brassica napus* (Chen et al., 2017; Scheben et al., 2020), and sesame (Yol et al., 2021). To our knowledge, this study is the first to demonstrate that ddRAD-Seq is a suitable approach to detect variants at a genome-wide scale in the sorghum genome. We discovered 1438 SNP markers dispersed over 10 LGs (**Figure 1**), perfectly matching the number of chromosomes in *S. bicolor* (n = 10), demonstrating the power of the method. Despite the average interval distance being sufficient for precise mapping (Wang et al., 2020b), there were a few> 10 cM gaps in our map, especially on LG7 (**Figure 1**). Such gaps are occasionally observed in linkage maps in cases such as the mapping population being generated from two parents whose genetic backgrounds are similar in some regions, or due to divergence from the reference genome (Wang et al., 2020b). The total length of our map was 1349.72 cM, while the shortest and longest LGs were 77.46 and 176.36 cM respectively. Our results were compared with those by de Souza et al. (2021), who developed genetic maps for sorghum with GBS, which were similar in length (1368.8 cM) but with greater variance in SNP distribution across the LGs. A comparable GBS method was also used by Somegowda et al. (2022) to construct a genetic map of 1269.9 cM in length, with an average distance of 2.22 cM between adjacent markers. Jin et al. (2021) constructed a high-density genetic map with a length of 1191.7 cM with the single-digest RAD-seq method and the average distance between markers was 1.11 cM. In summary, the linkage map produced here is of very similar length to previously published maps for sweet sorghum, but slightly better in marker distribution and density.

One notable difference from the current work is that all of these previous studies used RILs, whereas the current genetic map was generated from the F_2_ lines; different computational approaches were also used in each case. In a comparable study to ours, using ddRAD-seq in a *Brassica napus* F_2_ population, the resulting linkage map (3981.3 cM) was noted to be significantly longer than maps generated using *B. napus* RILs (Scheben et al., 2020). It was suggested that the heterozygosity of F_2_ lines can lead to genotyping errors, inflating the length of LGs; however, in our study the map length was consistent with those generated from RILs, suggesting that any such genotyping errors were successfully filtered out during SNP selection and linkage mapping. We did observe some differences in marker order between the genetic map and the reference genome, especially in the peri-centromeric regions, as well as two putative translocations. While the type of mapping population, number of individuals (Li et al., 2017), choice of parental lines and sequencing methods (Yol et al., 2021) might all cause differences in mapping, the high density and consistency of marker order across the large majority of the ddRAD-seq linkage maps presented here make them useful for localizing QTLs (Scheben et al., 2020). Furthermore, the QTLs identified below were all in regions for which the marker order was closely conserved with the reference genome, indicating that they are likely to be consistent with other sorghum genotypes.

A total of ten QTLs related to bioenergy traits were determined in our population, and PVEs ranged from 8.6% to 35.2% across the environments. Three major QTLs (q*PH*1.1, *qPH*7.1 and *qPH*9.1) associated with PH were found on chromosomes 1, 7, and 9. These QTLs had additive effects that ranged from 13.64 to 29.33 and dominance effects that ranged from -2.23 to 14.36. Previously, four dwarfism genes influencing PH in sorghum, referred to as *Dw*1, *Dw*2, *Dw*3, and *Dw*4, were described (Quinby and Karper, 1954; Upadhyaya et al., 2013). GA2-oxidase is encoded by the gene *Dw*1 (Sobic.009G230800; Chr09:57093313–57095643) (Yamaguchi et al., 2016); overexpression of GA2-oxidase lowers active GA levels and shortens plant height (Wang et al., 2020c). *Dw*2 (Sobic.006G067700; Chr06:42803037–42807134) encodes a protein kinase that is homologous to KIPK, a member of the AGCVIII subgroup protein kinase family which affects endomembrane function and cell division during the formation of sorghum internodes (Hilley et al., 2017; Olivera et al., 2020). The *Dw*3 (Sobic.007G163800; Chr07:59821905–59829910 (Multani et al., 2003) gene is located on chromosome 7 and encodes an ABCB1 auxin efflux transporter. Other recently reported dwarfism genes include *Dw*5, found in a mutagenesis library of BTx623 (Chen and Laza, 2019), which affects sorghum plant height similarly to *Dw*1-*Dw*4 and decreases plant height by 36-53% (Habyarimana et al., 2020), and similarly *Dw7a* (Sobic.007G137101) can reduce sorghum height by 40–50% (Hashimoto et al., 2021). However, no protein-coding gene has yet been identified for *Dw4* (Hashimoto et al., 2021). In present study, the SNP identified at Chr7_59610235 on QTL *qPH*7.1 had the largest contribution (34.8%) to PH and is located 220 kb away from the *Dw3 a priori* candidate gene (Multani et al., 2003). A second putative SNP at Chr7_59803007 was identified 30 kb away from *Dw*3 (**Table 8**). Various studies have also presented QTLs controlling plant height in sorghum near this location (Kajiya-Kanegae et al., 2020; Faye et al., 2021) (**Table 8**). Moreover, we found that the major QTL associated with plant height on chromosome 7, located between 83.204–107.873 cM, overlapped with two smaller QTLs for other bioenergy-related traits, Brix, and fresh biomass weight. This region also contains the *Dw*3 gene, and while it is possible that this gene also regulates biomass through limitation of plant height, the presence of the additional QTL for Brix suggests that this region may be a hot spot for genetic control of bioenergy production in sorghum. We also mapped *qPH*9.1 (Chr9_3610075) on chromosome 9 across both environments, but this QTL is distinct from the previously reported *Dw*1 gene (Sobic.009G230800, Chr09:57093313–57095643) (Hilley et al., 2016; Yamaguchi et al., 2016). It may demonstrate the presence of another candidate PH gene because QTL overlapping with *qPH*9.1 were also mapped to this position in a study by Wang et al. (2014). When we evaluated the environments individually, *qPHA*4.1 was colocalized with previous QTLs (Phuong et al., 2013; Zhang et al., 2015) on chromosome 4, *qPHA*9.2 was determined to be 33 kb away from *Dw1* on chromosome 9 (Hilley et al., 2016).

**Table 8:** QTL regions and SNPs associated with genes published in previous studies.

Traits	QTLs	SNPs	Related Genes	Previous Studies
**PH**	*qPHA4.1*	Chr4_5337376		Phuong et al., 2013 Zhang et al., 2015
	*qPH*7.1	Chr7_57913968 Chr7_64806414	*Dw*3 (Chr07:59821905-59829910)	Multani et al., 2003
				Kajiya-Kanegae et al., 2020
				Faye et al., 2021
	*qPHA*9.1	Chr7_ 57059477	Dw1 (Chr09:57093313-57095643)	Hilley et al., 2016
**PJ**	*qPJ*6.1	Chr6_50628405	*D* (Chr06:50896169-50898604)	Zhang et al., 2018 Mocoeur et al., 2015 Burks et al., 2015 de Souza et al., 2021 Ji et al., 2022

The stalk sugar yield of sweet sorghum, which is influenced by sugar content and stalk juice, determines its potential for ethanol production (Guan et al., 2011). Although stem juice yield is a key factor in this regard, limited studies have been conducted to identify related genes/QTLs (Murray et al., 2008a; Murray et al., 2008b; Guan et al., 2011). In this study, one major QTL (*qPJ*6.1) for PJ was determined on chromosome 6, which explained 35.2% of the phenotypic variance. In recent years, a few studies have reported QTLs associated with PJ in sorghum that overlap with this position (de Souza et al., 2021; Ji et al., 2022). Mocoeur et al. (2015) identified a QTL with a high PVE (37.5%) located on chromosome 6 (48787617-50907017) associated with juice yield. Moreover, Burks et al. (2015) also determined a QTL associated with juice yield, and this QTL had a significant association with midrib color, sugar yield, sugar yield by weight, moisture, juice volume, and juiciness. These two QTLs also were found to colocalize with our QTL, *qPJ*6.1, on chromosome 6. Moreover, the *qPJ*6.1 QTL for PJ was located at 267 kb from the *D*-gene (Sobic.006G147400, which encodes a plant-specific NAC transcription factor that controls the juiciness of the stem (Zhang et al., 2018). Surprisingly, the positive effect on juice content at *qPJ6*.1 was associated with the allele from the ‘low-juice’ parent (Ogretmenoglu). This reflects the fact that juice yield is a complicated quantitative trait linked to plant height, biological yield, and sugar content (Burks et al., 2015; Han et al., 2015); it can be assumed that one of these other traits masks the effect of the beneficial *qPJ6.1* allele in Ogretmenoglu. We also mapped four major QTLs associated with FBW; one of them, *qFBW*6.1 (Chr6_39024006), had a PVE of 14.5% and can be considered a novel QTL, because it did not overlap with any previously determined QTLs on chromosome 6.

Common QTLs determined across more than one environment or population are defined as stable QTLs, which may be more useful in breeding (Qu et al., 2021). Using variance analysis, the G × E interaction is determined as a QTL-by-environment (Q × E) interaction in QTL mapping studies (Chen et al., 2010). In the present study, variance analysis revealed that environmental effects play an important role in the development of bioenergy-related traits, especially for PH. Ten QTLs in total and three QTLs for PH were identified from combined data from two environments, including both lowland and highland sites. During the vegetative period, the lowland site had a much higher maximum temperature (39.7-40.9°C) than highland (33.0-34.9°C), and very little rain (**Table 1**). In this period, the optimal temperature range for sorghum is 26-34°C (Hammer et al., 1993). This situation may have caused variation in bioenergy-related traits; therefore, the QTLs detected across both environments are more likely to be stable under different conditions, although this should be confirmed with additional phenotyping data from different years. This was supported by the finding of two plant height major QTLs (*qPHA*4.1 and *qPHA*9.2) that were only evident when data from the two environments were analyzed individually, and do not co-localize with those determined using combined data (**Figure 2**).

Phenotypic traits are highly influenced by both environmental changes and growth stage. Therefore, molecular markers should be integrated into breeding programs to allow desired traits to be selected rapidly and independently of environment effects. In the present study, CAPS markers closely linked with several QTLs for pH, BX, PJ, and FBW traits were developed. Two markers, PH1 and BX3, were positioned in the same region of chromosome 7 because of their overlapping QTLs. PH1, PH7, BX3, BX6, PJ13, PJ15, and FBW5 markers were each strongly linked to their respective QTL regions. Moreover, the PH1 marker was approximately 220 kb from the *Dw3* gene, while FBW5 was derived from the novel QTL (*qFBW*6.1) for FBW identified on chromosome 6. All markers, which were validated on eight lines and three cultivars, showed perfect matches with the detected SNPs. Although CAPS markers have been widely used in genetic analysis, there is only one study in which genome-specific CAPS markers have been developed in sorghum. Burow et al. (2019) developed CAPS markers to identify the genotype of bmr6-12 lines and determined that the GA 340 cultivar was classified as bmr6 while BTx623-bmr and B03288bmr were classified as bmr12. Several SNP markers closely linked with the eight major and two minor QTLs reported here can supply beneficial information to facilitate fine mapping of bioenergy-related traits. CAPS markers converted from these SNPs could also be readily used for pyramiding genes for bioenergy-related traits in breeding programs in the near future.

## 5 Conclusion

We used the ddRAD-seq approach to construct a high-density linkage map developed from an F_2_ population in sweet sorghum, and to screen the linkage map for bioenergy-related traits. Ten QTLs associated with plant height, Brix, fresh biomass weight, and plant juice were identified from combined data from two contrasting environments, including lowland and highland sites. In addition to the identified major QTLs, a novel QTL for effective fresh biomass weight and newly improved CAPS markers, one of which was from this region, add to the current knowledge about these traits and provide additional capacity for the improvement of bioenergy production in sorghum.

## Data availability statement

The datasets presented in this study can be found in online repositories. The names of the repository/repositories and accession number(s) can be found below: https://www.ncbi.nlm.nih.gov/, PRJNA894980.

## Author contributions

BU designed and supervised the experiments. EY, BU and SL contributed final editing of manuscript. BU analyzed the data of map and QTL. BG, EY, and BU conducted the experiments and analyzed the data. BG, EY, CE and BU conducted the field trials. All authors contributed to the article and approved the submitted version.

## Supplementary material

Supplementary Figure 1 Dot plot comparison of the genetic positions of SNPs in each linkage group (horizontal axis) with their mapped position on the corresponding chromosome in the reference genome (vertical axis)
