# Supplementary figures and images for: Construction of a high-density genetic linkage map and QTL mapping for bioenergy-related traits in sweet sorghum [Sorghum bicolor (L.) Moench]
Source: Front Plant Sci. 2023 Jun 5;14:1081931. doi: 10.3389/fpls.2023.1081931 (PMC10278949; doi:10.3389/fpls.2023.1081931)

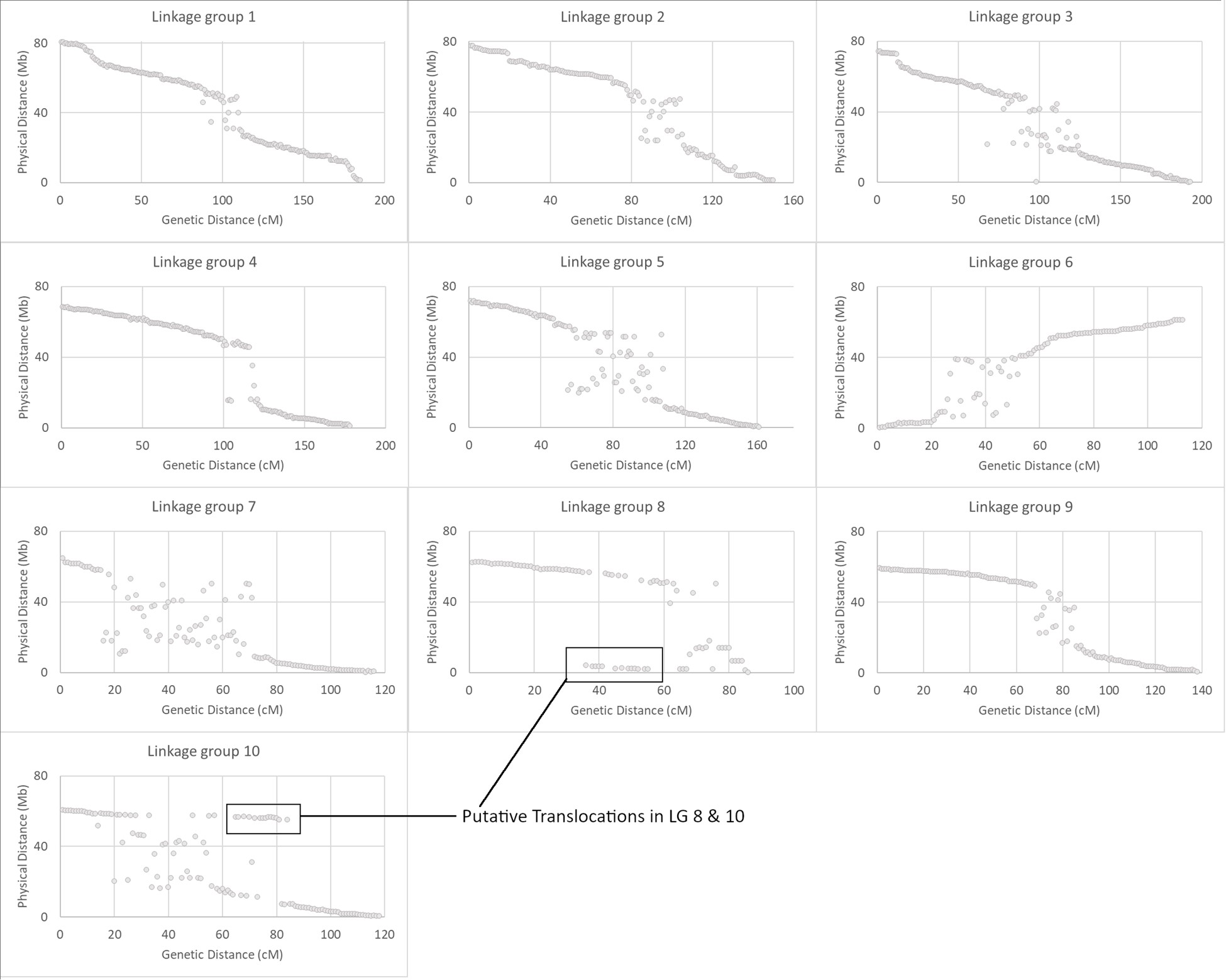

Supplement: Supplementary Figure 1 — Dot plot comparison of the genetic positions of SNPs in each linkage group (horizontal axis) with their mapped position on the corresponding chromosome in the reference genome (vertical axis) [file Image_1.jpeg]
